# Implicit Essentialism: Genetic Concepts Are Implicitly Associated with Fate Concepts

**DOI:** 10.1371/journal.pone.0038176

**Published:** 2012-06-07

**Authors:** Wren A. Gould, Steven J. Heine

**Affiliations:** Psychology Department, University of British Columbia, Vancouver, British Columbia, Canada; Ecole Normale Supérieure, France

## Abstract

Genetic essentialism is the tendency for people to think in more essentialist ways upon encountering genetic concepts. The current studies assessed whether genetic essentialist biases would also be evident at the automatic level. In two studies, using different versions of the Implicit Association Test [1], we found that participants were faster to categorize when genes and fate were linked, compared to when these two concepts were kept separate and opposing. In addition to the wealth of past findings of genetic essentialism with explicit and deliberative measures, these biases appear to be also evident with implicit measures

## Introduction

Genes play an influential role in human development by predisposing individuals toward particular outcomes in their lives. Media coverage of genes tends to overstate this influence, though. For instance, the media frequently reports on the strong causal influence that the DRD4 gene has on novelty-seeking behaviours [2], [3]. However, meta-analyses reveal that it’s relation with novelty-seeking yields is less than d = 10, suggesting that the relationship isn’t particularly strong and the evidence is all correlational [4], [5]. These instances may not only be examples of sensationalist media reporting. Instead, they may provide a glimpse of a potent cognitive bias–genetic essentialism.

Genetic essentialism is the tendency to think in more essentialist ways upon encountering genetic attributions [6]. Essences are the perceived pith that inheres within living creatures which makes them as they are [7], [8], [9], [10]. Insofar as underlying processes are difficult to observe, material placeholders may be recruited to better understand internal processes. Genes are especially appropriate essence placeholders because they are perceived as immutable, fundamental, homogenous, discrete, and natural [6]. Hence, when people encounter genetic attributions, they may view associated characteristics in more essentialist ways. Rather than seeing genes as simply factors that predispose individuals to particular outcomes, people often see genes as determining outcomes [11]. Although there are monogenic phenomena in which genes do have a deterministic relation with the phenotype (such as with Huntington’s disease), this is the exception. More commonly, multiple genes are probabilistically associated with a phenotype, and their expression is dependent upon environmental events. In particular, it is highly unlikely that psychological traits would be a function of just a few genes [12]. In these cases, genes do not have a deterministic relation with phenotypes, and it is inappropriate to assume that they do.

Viewing genes in fatalistic ways may have undesirable consequences [6]. For instance, people may see genes as absolving people of responsibility for their actions, and even of their crimes [13], [14]. Genetic explanations for group differences also reduce math performance for women [15], and can enhance stereotyping [11], [16].

Although it is theoretically possible that other concepts could also serve as essence placeholders, genes may be uniquely apt placeholders. Some researchers suggest that essentialism is a potent form of folk-biology, and that it derives from an evolved understanding of living kinds that was adaptive in our ancestral past; that is, essentialism may act as an adaptive mechanism to understand biological inheritance [17], [18]. Consistent with this theory, children reason in essentialist ways, expecting one’s rearing to be of little consequence for one’s innate behaviour [19]. To the extent that this is true, material biology may be an ideal placeholder for folk biological concepts. People may turn to biological matter as a ready instantiation of essences before recruiting other kinds of placeholders (e.g., socialization). Although there is some evidence that environmental influences, like socialization, may be interpreted as having an impact on the phenotype in essentialist ways [20], individuals more often appear to think of environmental influences as lying outside the self, and reflecting one’s choices [13], [15]. Because genes are so chronically associated with determined outcomes, other explanations may be seen as more linked to undetermined, chosen outcomes.

Since genes may non-arbitrarily relate to fatalistic outcomes, it seems plausible that the connection between implicit genetic perceptions and fate may be automatic, especially when compared to other implicitly perceived explanations, such as socialization. It is important to identify which implicit associations exist with genetic concepts in order to develop a rich understanding of the associative network that may predispose individuals to genetic essentialist biases. The vast literature on implicit cognition has focused on a variety of topics, such as implicit attitudes [1], [21], [22], implicit stereotypes [23], [24], [25] and automatic goals [26]. Thus far, the implicit associations that may result from essentialist ontologies have not yet been assessed. The current studies address this with a common tool used in assessing implicit social cognition–the Implicit Association Test [1].

### The Present Research

In our first study, we used the Implicit Association Test (IAT) to measure the automatic connections between genes and fate. The IAT has shown relatively robust test-retest reliability [27] and predictive validity [28], and has become a standard measure of implicit associations in the psychological literature.

The IAT asks participants to place words into one of two opposing categories, such as flowers vs. insects using keys on the left and right side of a keyboard. It then asks participants to do the same with two other opposing categories, for instance, the evaluative judgment good vs. bad. Participants then complete trials in which “flower” words are paired with “good” words, while “insect” words are paired with “bad” words, as quickly as they possibly can. They are then asked to do the reverse: pairing “flower” words with “bad” words and “insect” words with “good” words. If the mean response times (in milliseconds) diverge between these different trials, then there are likely automatic associations between these categories, such that participants may more quickly associate “flower” words with “good” words. Indeed, this is exactly what researchers have found in conducting a flowers vs. insects IAT [1].

The constraints of the IAT require dichotomies and contrasts. For the current study, “choice” emerged as a likely contrast for fate, since fates are inherently assigned and are not chosen. Presumably, choice can be understood as a completely undetermined act of free will [29]. Though some people view determinism and free will as peacefully co-existing [29], many philosophers and lay people construe determinism and free will as being incompatible, which legitimizes the inclusion as “choice” as an opposite of “fate” [30], [31]. We chose “socialization” as a clear opposite for genes, since nature (i.e. genes) and nurture (one’s socialization) have long been juxtaposed as alternative categories [32]. We expected that participants may pair “gene” words with “fate” words in the same way that they automatically pair “flower” words with “good” words, when choice and socialization are contrasted.

In Study 2, we adapted a single-target version of the IAT to look more closely at the associations between genes and fate vs. genes and choice. Since the IAT in Study 1 relies on dichotomies we cannot be certain that genetic associations with fate would be driving the effect. An alternative interpretation may be that socialization is particularly associated with choice, which might result in the particular differences in response times we found in this study. Study 2 disentangles this problem by using a single-target version of the IAT.

Like the original IAT, this single-target version has shown good reliability and validity [33]. Unlike the original, though, it relies on only one group of dichotomous categories. For instance, it pairs insect words with “bad” words while asking participants to place “good” words in a category of its own. Subsequently, it pairs insect words with “good” words while asking participants to place “bad” words in its own category. By comparing these two different trials, we can more carefully assess whether or not insects are more closely associated with good or bad evaluations. Thus, this task was ideal for assessing how closely genes were associated with fate, as compared to choice.

### Ethics Statement

For all of the studies presented, we obtained behavioral research ethics board approval from the University of British Columbia. Participant’s consent was indicated by clicking on a computer button, and thereby choosing to continue on to the rest of the study, as was approved by the UBC Behavioral Research Ethics Board.

## Method

### Study 1

Thirteen men and 34 women were recruited by Amazon.com’s Mechanical Turk, which compensates participants with Amazon.com merchandise in return for completing on-line tasks. Data recruited from Mechanical Turk have been shown to be comparable to data collected by more traditional, lab-based methods [34]. Participants’ ages ranged from 20 to 58 (*M* = 39), and the majority (*n* = 37) were of European descent. Two others were East Asians, one was Middle-Eastern, and the rest described themselves as “white” or as “multi-racial.” All were citizens of the United States.

Participants completed an IAT that was specifically adapted to capture associations between genes, socialization, fate, and choice. The words chosen for these categories were typically synonyms or near-synonyms for the category name, and so we expected they would be closely identified with each other. Participants were allotted practice trials to place genetic words (e.g., “genome,” “DNA,” “heredity”) vs. socialization words (e.g., “nurture,” “training,” “experience”) into their proper categories by typing corresponding keys on the keyboard. Afterwards, they practiced categorizing words relating to fate (e.g., “God,” “destiny,” “certainty,”) vs. words related to choice (e.g., “free-will”, “option”, “opinion”; the complete list of words in the respective categories is provided in Appendix S1). Then participants were asked to place “fate” words and “gene” words into the same category while placing “choice” and “socialization” words in a second category as quickly as possible. They were then directed to do just the opposite, placing “fate” and “socialization” words into the same category while identifying “choice” and “gene” words into another category. By comparing the differences in response times between these trials, we can calculate the magnitude of implicit bias. Response times were analyzed with the most recent scoring algorithm [35]. By subtracting response time means per participant for the Gene + Fate/Socialization + Choice trial from the response time means per participant for the Gene + Choice/Socialization + Fate trial, while dividing by the standard deviation of each participants’ responses, this scoring algorithm produces a standardized measure of IAT bias.

### Study 2

One hundred and thirty-five participants completed this study, however the results of 7 participants were excluded because more than 10% of their responses fell below the cut-off of 300 ms recommended in IAT studies [35]. This left 87 women, 40 men, and 2 participants who did not specify their genders, who were all recruited by Mechanical Turk. Their ages ranged from 18 to 67 (*M* = 36), and the majority (*n* = 99) were of European descent. The rest were East Asian (*n* = 7), African (*n* = 6), South-east Asian (*n* = 2), Middle Eastern (*n* = 4), with nine who self-reported races as Hispanic (*n* = 2), Caucasian (*n* = 2), Native American (*n* = 1), American (*n* = 1), as mixed race (*n* = 1), or as not knowing (*n* = 2). Two declined to respond. Again, all participants were citizens of the United States.

For this study, we adapted a single-target Implicit Association Test (ST-IAT). With this ST-IAT, participants were asked to learn associations with the same genetic words as in Study 1 via practice trials. However, they were not asked to learn associations with “socialization” words as a contrast category. Participants were subsequently asked to learn associations with fate words vs. choice words, as in Study 1. Afterwards, participants participated in several critical trials. In the first critical trial, subjects placed genetic words in the same category as fate words, while “choice” words were partitioned in another category (a Gene + Fate/Choice trial). In the second critical trial, subjects placed genetic words in the same category as choice words, while fate words were partitioned into another category (a Gene + Choice/Fate trial). By comparing the response times between these trials, we can get a clearer sense of how genes are related to fate, as opposed to choice, without invoking a comparison to socialization. As before, we adopted the scoring algorithm most often used in analyzing IAT data [35], which computes an individual, standardized measure of one’s bias.

Participants also completed two explicit measures of genetic determinism: the Belief in Genetic Determinism Scale [11] and the Genetics, Disease, & Stigma Survey [36].

## Results

### Study 1

We used a one-sample t-test to assess whether or not the standardized differences between the trials per participant generally differed from zero. Again, if the standardized differences in response times between the trials were different from zero, there would be evidence that it was easier than for participants to pair genes with fate and socialization with choice than vice versa. The results confirmed our hypothesis, *t*(46) = 8.24, *p*< 001. The average D-score was substantial (*M* = 0.43, *SD* = 0.34), and the difference between the mean for the two trials was 320 ms. Participants were slower to place gene words in the same category as choice words while placing socialization words with fate words, when compared to placing gene words with fate words and socialization words with choice words. Thus, this is evidence that participants are quicker to see genes as linked to fate when socialization is simultaneously associated with choice, rather than vice versa.

### Study 2

Again, because Study 1′s effects relied on associations with socialization, Study 2 was designed to minimize concerns that linkages with socialization drove effects. If participants were faster to place genes and fate into the same category, while relegating choice to another category, than they were to place genes and choice in the same category when placing fate in its own category, then this would be evidence that participants implicitly associate genes with fate more than choice. We conducted a one-sample t-test on participant’s standardized response time difference between trials, to see if a consistent bias arose on this standardized difference measure. Indeed, participants showed a significant bias (*M* = 0.13, *SD* = 0.36), such that they responded more quickly when genes were related to fate and “choice” was considered separately, *t*(128) = 4.26, *p*<001. The bias was smaller than observed in Study 1, though this may be expected since we did not contrast genes with socialization. The average participant responded to the Gene + Fate/Chance trial 133.76 ms faster than they responded to the Gene + Chance/Fate trial. Curiously, participants’ standardized difference scores were uncorrelated with either of the explicit measures – the Belief in Genetic Determinism Scale (α = .90), *r* = .02, *p* = 79, and the Genetics, Disease, & Stigma Survey (α = .74), *r* = −.03, *p* = .75, which is consistent with the notion that these attitudes are operating outside of conscious control. Additionally, the two explicit scales correlated moderately with each other, *r* = .59, *p*<.001, demonstrating convergent validity.

Study 2 confirms that genes and fate are more closely linked than genes and choice. Thus, the findings in Study 1 were likely driven in part by an association between genes and fate. This is evidence that the automatic connection between genes and fate is stronger than its connection with opposing constructs, which may bias more deliberative judgments about the properties of genes.

## Discussion

Over two studies, people implicitly associated genes with fate more than they did with choice. These implicit associations can help explain the essentialist reactions that people show when encountering genetic concepts [11], [15], [16]. People may form genetic essentialist associations implicitly, which may influence their explicit thoughts about these concepts as well.

It is unclear whether learning about genetic concepts contributes to implicit associations, or whether implicit genetic essentialism is universal. The cross-cultural and developmental evidence for essentialism suggest that people may be universally predisposed to think in essentialist ways, although studies of genetic essentialism have largely been limited to Western adult populations. It would be fruitful to explore how children in different cultures conceptualize genetic concepts when they first learn them.

The conclusions that can be drawn from these studies are restricted, though, due to limitations. First, because we exclusively used IAT-like tasks, our results relied on the dichotomies inherent in the IAT. Although people implicitly associate genes with fate words more than choice, it is quite possible that genes may not prompt fate-like constructs on their own. Also, it is unclear how automatic bias interacts with controlled genetic essentialist beliefs. In the current study, automatic genetic essentialist attitudes were uncorrelated with explicit attitude measures. This finding diverges from the low, but significant, correlations with explicit measures found in other IAT research. This suggests that these implicit genetic attitudes may have few consequences for explicit attitude measures. This is an important consideration with regard to the scrutiny over what the IAT exactly measures. For instance, if these studies assess the implicit activation of cultural ideas and attitudes [37], they may not impinge upon explicit attitudes, and their implications for participant’s own behaviour are less clear. More research is necessary to disentangle exactly what these implicit attitudes may mean for cognition and behaviour. Nonetheless, understanding how genetic concepts are associated with essentialist thinking is aided by this demonstration that people automatically associate fate words with genetic concepts more readily than they do choice words.

## Supporting Information

Appendix S1 **The complete list of words in respective categories is provided.**
(DOCX)
