# Supplementary material for: Implicit Essentialism: Genetic Concepts Are Implicitly Associated with Fate Concepts
Source: PLoS One. 2012 Jun 7;7(6):e38176. doi: 10.1371/journal.pone.0038176 (PMC3369887; doi:10.1371/journal.pone.0038176)
Supplement: Appendix S1 — The complete list of words in respective categories is provided. (DOCX) [file pone.0038176.s001.docx]

**Appendix A**

| **Choice-Based Words**  Free-Will  Decision  Option  Selection  Freedom  Preference  Opinion | **Fate-Based Words**  God  Plan  Necessity  Permanence  Blueprint  Destiny  Certainty |
| --- | --- |
| **Gene-Based Words**  Genome  DNA  Double-Helix  Cell  Bloodline  Heredity  Chromosome | **Socialization-Based Words**  Learn  Lifestyle  Nurture  Tradition  Training  Experience  Develop |
